# Impulse control disorders and dopamine agonists

**DOI:** 10.1192/j.eurpsy.2021.1268

**Published:** 2021-08-13

**Authors:** D. Martins, R. Faria, M. Pinho, S. Rodrigues

**Affiliations:** 1 Department Of Psychiatry, Hospital de Magalhães Lemos, Porto, Portugal; 2 Department Of Child And Adolescent Psychiatry, Centro Hospital e Universitário do Porto, Porto, Portugal

**Keywords:** Impulse control disorders, dopamine agonists, pathological gambling

## Abstract

**Introduction:**

Impulse control disorders (ICDs) are an adverse effect of dopamine agonists (DAAs) that affects the quality of life and can lead to legal, criminal and familiar problems.

**Objectives:**

Presenting a review of the mechanisms, prevalence and factors associated with the development of an ICD due to DAA use.

**Methods:**

Search on Pubmed database with combination of the following keywords were used: “Impulse control disorders”, “dopamine agonist” or “therapy”. We focused on data from studies published between 2015 and 2020. The articles were selected by the author according to their relevance.

**Results:**

DAAs are mainly indicated in the treatment of Parkinson’s Disease (PD), and are also used on symptoms of restless legs syndrome (RLS) and prolactinoma or lactation inhibition. Dopamine replacement therapy act on dopamine receptors in the nigrostriatal and the reward pathways, which plays a role in addictive behavior. The prevalence of ICDs ranged from 2.6 to 34.8% in PD patients and a lower prevalence in RLS patients. Some of the ICDs reported were pathological gambling, hypersexuality, compulsive shopping, obsessive hobbying, punding, and compulsive medication use. The factors associated with the development include the type of DAAs, dosage, male gender, younger age, history of psychiatric symptoms, earlier onset of disease, longer disease duration, and motor complications in PD.

**Conclusions:**

Further studies are needed to clarify the pathophysiology of the ICD in DAA therapy and determinate premorbid risk factors. The percentage of patients with ICDs is underrated, so it’s important to improve the patient’s evaluation, using validated and consensual assessment tools.

